# A study of microRNAs as new prognostic biomarkers in anal cancer patients

**DOI:** 10.2340/1651-226X.2024.27976

**Published:** 2024-06-20

**Authors:** Olav Dahl, Mette Pernille Myklebust

**Affiliations:** a Department of Oncology, Haukeland University Hospital, Bergen, Norway; b Department of Clinical Science, The Medical Faculty University of Bergen, Bergen Norway

**Keywords:** Prognostic MicroRNAs, anal cancer, clinical study, new biomarkers

## Abstract

**Background:**

MicroRNA (MiR) influences the growth of cancer by regulation of mRNA for 50–60% of all genes. We present as per our knowledge the first global analysis of microRNA expression in anal cancer patients and their prognostic impact.

**Methods:**

Twenty-nine patients with T_1-4_ N_0-3_ M_0_ anal cancer treated with curative intent from September 2003 to April 2011 were included in the study. RNA was extracted from fresh frozen tissue and sequenced using NGS. Differentially expressed microRNAs were identified using the R-package DEseq2 and the endpoints were time to progression (TTP) and cancer specific survival (CSS).

**Results:**

Five microRNAs were significantly associated with 5-year progression free survival (PFS): Low expression of two microRNAs was associated with higher PFS, miR-1246 (100% vs. 55.6%, *p* = 0.008), and miR-135b-5p (92.9% vs. 59.3%, *p* = 0.041). On the other hand, high expressions of three microRNAs were associated with higher PFS, miR-148a-3p (93.3% vs. 53.6%, *p* = 0.025), miR-99a-5p (92.9% vs. 57.1%, *p* = 0.016), and let-7c-3p (92.9% vs. 57.1%, *p* = 0.016). Corresponding findings were documented for CSS.

**Interpretation:**

Our study identified five microRNAs as prognostic markers in anal cancer. MiR-1246 and microRNA-135b-5p were oncoMiRs (miRs with oncogene effects), while miR-148a-3p, miR- 99a-5p, and let-7c-3p acted as tumour suppressors in anal cancer patients.

## Introduction

Anal cancer is a rare disease at each cancer center, but 27 000 patients are diagnosed with this cancer globally each year, and the incidence is rising [1, 2]. The treatment is based on clinical and radiological examinations as the basis for staging according the TNM system [3, 4]. The standard treatment is radiation combined with mitomycin C or cisplatin and a pyrimidine (5-fluorouracil (5-FU) or capecitabine) with surgery as salvage treatment [4, 5]. More than 90% of the tumours are associated with human papilloma virus (HPV) infection and the prognosis is worse for higher viral loads [6, 7]; however, the HPV negative tumours still have the poorest prognosis [8, 9]. Tumour control is usually achieved for the early stages, but the locally advanced tumours have a relatively poor prognosis with about 50% long-term survival [3]. Despite being easily accessible for tissue sampling, the genetic landscape of anal cancer is not well-characterized.

It is now recognized that microRNAs (miRs) influence the growth of cancer by regulation of messenger RNA (mRNA) for 50–60% of all genes, serving as oncogenes (oncoMiRs) or tumour suppressor microRNAs (suppressor MiRs) [10]. We have earlier shown that miR-15b modulates the cell cycle regulation by HPV stimulation of E2F in anal cancer [11]. To our knowledge, the present study is the first global analysis to decide the role of microRNA expression in anal cancer patients.

## Material and methods

### Patients

Anal cancer patients were recruited at the Department of Oncology, Haukeland University Hospital, from September 2003 to April 2011. We included 28 patients with squamous cell cancer and one with cloacogenic cancer, but adenocarcinomas were excluded. A total of 31 patients were included with the median age of 63 years (range: 28–87). Two patients were excluded due to insufficient RNA quality, leaving nine men and 20 women for analysis. For the eligible patients, the primary tumour was localized in the anal canal in 19 patients and in the perianal region in the remaining 10 patients. The clinical workup included proctoscopy, computed tomography (CT) of chest, and abdomen and magnetic resonance imaging (MRI) of the pelvic area. The patients were originally classified according to the TNM 4^th^ edition [12], which has T and N classification almost as identical as the TNM 8^th^ edition [3, 13]. Skin squamous cell cancers within 5 cm from the anal verge were also treated as anal cancers, in accordance with the TNM 8^th^ classification. The T categories are shown in Table 1. For stage classification, we used the TNM 7^th^ edition [14]. Fourteen patients had no nodal spread, and 15 had nodal spread, but none had distant metastases. The patients were treated according to Nordic Anal Cancer Group (NOAC) protocols 3, 5, and 7; all clinical data were drawn from the patient’s primary journals [5, 15]. Radiation dose to the primary tumour and involved nodes varied between 54 and 60 Gy according to stage. In early stages, one patient was treated with radiation alone (54 Gy) and one patient had surgery alone; none of these had a recurrence. Lymph node regions without evidence of tumour tissue received a radiation dose of 42–46 Gy, except for one T_1_N_0_ tumour where elective inguinal lymph node irradiation was omitted. Chemotherapy was given either as one or two courses of 5-FU and mitomycin C concurrent with the radiation or cisplatin and 5-FU were given as two cycles prior to radiation and the third cycle concurrent with radiation (10 patients).

**Table 1:** Patient characteristics for the 29 patients where pre-therapy biopsies were analysed for microRNA expression.

**Sex**	
Male	9
Female	20
**Age**	
Years	63.0 (range 28.0–87.0)
**Location**	
Anal canal	19
Anal margin	10
**Size**	
Median (cm)	5.5 (range 1.9–12.0)
**T-stage**	
T1	1
T2	13
T3	8
T4	7
**N-stage** (TNM 4)	
N0	14
N1	6
N2	5
N3	4
**Stage** (TNM 7)	
I	1
II	11
III A	7
III B	10
IV	0
**Treatment**	
RT and FuMi	17 (*2 also surgery)
RT and CiFu	10
RT alone	1
Surgery alone	1
**High-Risk HPV**	
Positive	23
Negative	6

TNM classification according to 4^th^ edition, stage according to 7^th^ edition. Chemotherapy was administered according to the standard combination of fluorouracil and Mitomycin C (FuMi) and cisplatin and fluorouracil (CiFu).

The follow-up was scheduled every 6 months for 5 years by an oncologist or a surgeon with clinical examination, rectoscopy if feasible, blood counts, chest X-ray, and ultrasound of the abdomen with additional CT or MR examination when clinically indicated. The median follow up was 7 years (range 0.5–14.5 years) with one third alive at 10 years follow-up.

### Biopsies and RNA extraction

Biopsies from anal cancer patients were flash frozen in liquid nitrogen and stored at -80°C until further processing. For RNA extraction, we used the AllPrep DNA/RNA/miRNA Universal Kit (Qiagen PN 80224). Briefly, approximately 20 mg of frozen tissue was disrupted and homogenized in 600 µL RLT lysis buffer by TissueLyzer LT (Qiagen) at 50 Hz for 7 min. Further, the homogenized lysate was processed according to the manufacturer’s protocol, including an on-column DNase digestion (RNase-Free DNase Set, Qiagen# 79254). Total RNA was eluted in 50 µL nuclease-free water. Total RNA was quantified by the OD260 on the NanoDrop2000 spectrophotometer, and the quality was assessed using Agilent RNA 6000 Nano Assay using Agilent Bioanalyzer. The RNA samples were stored at −80oC.

### MicroRNA sequencing

Libraries were prepared from 100 ng total RNA using the QIAseq miRNA Library Kit (Qiagen). Adapters containing UMIs were ligated to the RNA before converting RNA to cDNA. Amplification consisted of 16 polymerase chain reaction (PCR) cycles. Library quality control was performed using the TapeStation 4200 (Agilent) prior to pooling in equimolar ratios and sequencing on a NextSeq500 instrument at Qiagens Sequencing Facility (Hilden Germany). Raw data were de-multiplexed and FASTQ files were generated using the bcl2fastq software (Illumina Inc.). The quality of the FASTQ files was assayed using the FASTQC tool. Annotations of the obtained sequences were conducted using GRCh37 and miRbase20 as references. Adapter sequences were trimmed using Cutadapt (1.11), and reads were mapped using Bowtie (2.2.2). For aligning reads to miRbase, the criterion was to have a perfect match to the reference sequence. Regarding mapping to the genome, one mismatch was allowed in the first 32 bases of the read sequence. No indels were allowed. In total, 14.2 million reads were obtained for each sample.

### Differential expression analysis

The count matrix was then globally analyzed using the R-package DEseq2 with mean expression related to complete clinical response (cCR) to primary treatment versus residual tumours with fold change (FC) above 1.5 (or below 0.67) with an uncorrected *p*-value < 0.05, and a similar procedure using the endpoint progression or recurrent disease against no evidence of disease (NED) also with FC < 0.67 or FC > 1.5 and *p*-value < 0.05 [16]. The microRNAs with rowSum (total row count for all samples) above 150 in at least one group leaving out very lowly expressed microRNAs, were then selected for final analysis of time to progression or recurrence during follow-up (TTP), and cancer-specific survival (CSS) using IBM SPSS 26 package (IBM Corp., Armonk, NY, USA). The number of MiRs loaded into the DEseq2 analysis was 1012 and 22 were identified as differentially expressed.

### Target enrichment of MiRs

Experimentally validated targets for the five MiRs found to be prognostic markers were identified using the interactive web tool MIENTURNET [17] with targets from the miRTarBase [18]. The thresholds in the MIENTURNET enrichment analysis tool were set to (1) minimum one miRNA-target interaction and (2) adjusted *p*-value (FDR) 0.15. The list of identified targets was imported into the R-package clusterProfiler for enrichment analysis and graphic results production [19]. Graphical figures were produced and edited using Affinity Designer (version 1.10.1.1142., Serif).

### HPV testing

HPV was detected using the HPV (High Risk) TaqMan PCR Kit (Norgen Biotek Corporation, PN TM32200) according to the manufacturer’s protocol. The kit detects HPV types 16, 18, 31, 33, 35, 39, 45, 51, 52, 56, 58, 59, and 68. Briefly, 3 µL of patient DNA was added to a total volume of 17 µL MDx TaqMan 2X PCR Master Mix, nuclease free water, and 2 µL HPV (High Risk) Primer Probe Mix. The PCR conditions were 95°C for 3 min for denaturation, followed by 40 cycles of 95°C for 15 s and 60°C for 30 s. LightCycler 480 using the LightCycler v.1.5 software was used for detection. Positive and negative controls were included as per instructions from the manufacturer. A resulting Cq-value < 35 with exponential amplification curves for HPV and internal control was considered positive.

### Statistics

The primary endpoint TTP was defined as the time from biopsy date to residual progressive tumour after therapy or diagnosis of local or distant recurrences presented in the figures as progression free survival (PFS). For calculation of CSS, death as a result of anal cancer was recorded as an event and survival was censored at death as a result of any cause other than anal cancer or end of follow-up. Deaths as a result of anal cancer or complication to therapy were marked as an event, deaths due to other causes including cancers were censored. Survival was estimated by the Kaplan Meier actuarial method, and differences were tested by the log-rank test using IBM SPSS 26. A two-tailed *p*-value below 0.05 was considered statistically significant.

The expressions of the microRNAs and HPV were dichotomized as low below and high above the median value, and correlations tested by the Chi-square test.

The study received ethics approval from the Regional Ethics Committee (REK IV Sak200/2000) and all patients gave their written informed consent to participate.

## Results

A total of five patients (17%) had as expected uncertain residual local disease at follow-up 1–3 months after primary radiation and chemotherapy. Progression was observed in seven of 29 anal cancer patients (Local progression 2, local and distant 2, pulmonary metastases 1, multiple sites 2). Five deaths were a result of anal cancer and one occurred due to complication of therapy for recurrence.

High-risk HPV was documented in 79% of the patients (Table 1), with four male and two female patients having HPV-negative tumours. Male sex was associated with recurrence (*P* = 0.016), but not initial clinical response (*P* = 1.00). Three of six patients with HPV negative tumours, and only three of 23 HPV positive tumours progressed. The PFS at 10 years in HPV positive patients was 87.5% (95% confidence interval [CI]: 74.2–100) and 50.0% (95% CI: 10.0–90.0) in HPV-negative patients (*p* = 0.09), respectively. HPV expression was not associated with high or low expression of any of the microRNAs with prognostic impact as analyzed by the Chi-square test.

Twenty-two unique microRNAs satisfied the expression criterion for initial cCR, and eight microRNAs satisfied the criterion for association with recurrence. All these microRNAs were further analyzed for their prognostic impact using Kaplan Meier analyses for TTP and CSS.

### MiR-1246

**Figure 1:**
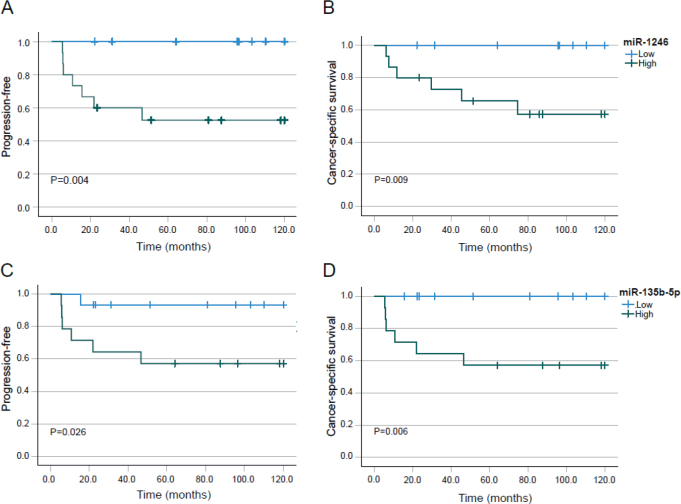
Progression free survival (PFS) and cancer specific survival (CCS) for miR-1246 (A and B), microRNA – 135b-5p (C and D). The Kaplan–Meier curves show the effect of expression of each microRNA above (High) or below (Low) the median expression in anal cancer tissue.

MiR-1246 was 2.4-fold higher in patients with residual tumours or relapses than those without any residual tumour or relapse after chemoradiation. MiR-1246 was not significantly differently expressed in relation to T stage or N stage. However, MiR-1246 was significantly higher expressed in men, eight with high expression and only one in the low group, versus for women who 12 had low expression and eight high expressions (*p* = 0.020). The PFS for patients with expression below median was 100% at 10-years follow up, in contrast to those with higher expression where PFS at 10-years was 55.6% (95% CI: 30.9–80.3), *p* = 0.004, see Figure 1A. The corresponding values for CSS were 100% at 10-years with low expression and 67.7% (95% CI: 46.1–89.3) at 5-years and 60.2% (96% CI: 35.1–85.3) at 10-years follow up, *p* = 0.009 (Figure 1B).

### MiR-135b-5p and miR-135b-3p

The global analysis revealed a 2.8-fold higher expression of MiR-135b-5p in patients with residual tumours or recurrence compared to those where the cancer was controlled by radiation combined with chemotherapy. MiR-135b-5p expression was marginally higher expressed in T_3-4_ tumours compared with T_1-2_ tumours (*p* = 0.049), but there was no association to N-stage or sex. Of those with low expression only one local recurrence was observed after 1.3 years, with 10-year PFS of 92.3% (95% CI: 80.4–100) and for those above median expression 10-year PFS was 59.3% (95% CI: 34.0–84.6), *p* = 0.026, Figure 1C. The corresponding CSS for low expression of MiR-135b-5p was 100% at 10-years, and for high expression it was 57.8% (95% CI: 31.7–83.9) at 5 and 10-years, (*p* = 0.006, Figure 1D).

### MiR-148a-3p

**Figure 2:**
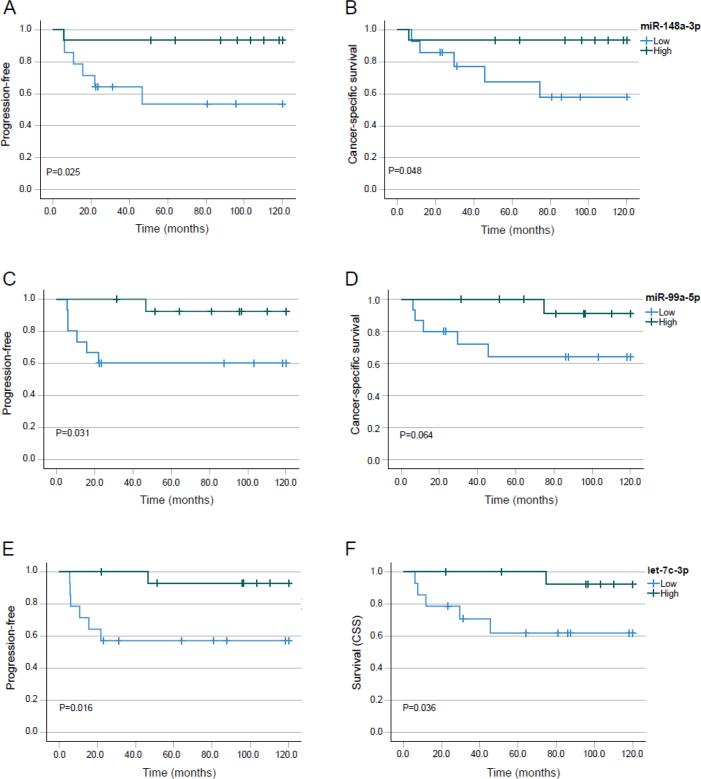
Effect on PFS (A, C, and E) and CSS (B, D, and F) for miRs identified as tumour suppressors. MiR-148a-3p (A and B), miR-99a-5p (C and D), and let-7c-3p (E and F) in low versus high expression groups. The threshold for categorization as ‘Low’ or ‘High’ was the median expression for the specific miR.

MiR-148a-3p was significantly higher expressed in early tumours, T_1-2_, than the advanced tumours T_3-4_, *p* = 0.005. There was no relation of expression to N-stage or sex. For patients with high tumour expression of MiR-148a-3p PFS at 10 years was 93.3 % (95%CI 80.8-100) and 53.6% (95%CI 25.4-81.8) for those with low expression, *p* = 0.025 (Figure 2A). The corresponding CSS were 92.3% (95%CI 77.8-100) and 42.1% (95%CI 7.4-76.8), respectively, *p* = 0.048 (Figure 2B). These results indicate that miR-148a-3p is a tumour-suppressing microRNA.

### MiR-99a-5p

The expression of MiR-99a-5p was 2.1-fold higher and for MiR-99a-3p 2.5-fold higher in the group with complete response compared to the group without complete response after radiochemotherapy. The levels of MiR-99a-5p and miR-99a-3p were not related to T-stage, N-stage, or sex. High expression of MiR-99a-5p was associated with 10-year PFS of 92.9% (95% CI: 79.4–100) versus 57.1% (95% CI: 31.2–83) for the low expression group, *p* = 0.016 (Figure 2C). For CSS, high expression of MiR-99a-5p was associated with 10-year survival of 88.9% (95% CI: 68.3–100), and for low expression 10-year CSS was 53.6% (95% CI: 20–84.2), *p* = 0.038 (Figure 2D).

### Let-7c-3p

Let-7c-3p was significantly higher expressed in lower stages (T_1-2_) compared to higher stages (T_3-4_), four patients in the low let-7c-3p group and 10 with high let-7c-3p in lower stages versus 10 with low expression and five with high expression in advanced stages, *p* = 0.040, but there was no relation of expression and N stage or sex. PFS at 10-years was 92.9% (95% CI: 79.4–100) with high expression and 57.1% (95% CI: 31.2–83.0) (*p* = 0.016) with low expression of let-7c-3p (Figure 2E). The corresponding values for CSS at 10-years were 92.3% (95% CI: 55.8–100) and 61.9% (95% CI: 35.2–88.6), *p* = 0.036, respectively (Figure 2F). The data indicate a tumour suppressive role of Let-7c-3p.

## Functional enrichment

**Figure 3:**
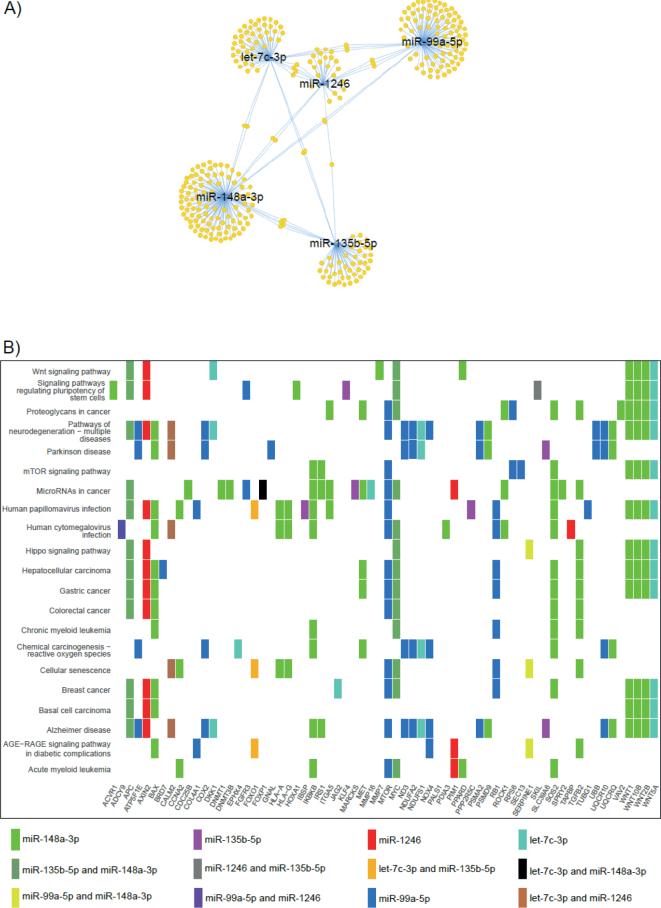
Target analysis of the prognostic microRNAs. (A) Network showing common targets among the microRNAs in the analysis. (B) Pathway analysis of the prognostic miRs showing the KEGG pathways identified as enriched. The miRs enriched in each pathway are indicated.

To explore the biological function of the five MiRs identified as prognostic markers, their most prominent targets were identified in the miRTar database. Schematic network of the targets and enriched target KEGG pathways are shown in Figure 3. Our analysis identified enrichment for proteins in several KEGG pathways associated with other malignancies, for example, microRNAs in cancer (hsa05206), hepatocellular carcinoma (hsa05225), gastric cancer (hsa05226), colorectal cancer (hsa05210), breast cancer (hsa05224), basal cell carcinoma (hsa05217), and myeloid leukaemia (hsa05220 and hsa05221). Two pathways associated with virus infection were found to be enriched HPV infection (hsa05165) and human cytomegalovirus infection (hsa05163). Targets in the Wnt signalling pathway (hsa04310), Hippo signalling pathway (hsa04390) and mTOR signalling pathway (hsa04150) were also enriched.

## Discussion

The present study investigated the expression profile of microRNAs in anal cancer and possible prognostic micoRNAs were identified. High expressions of MiR-1246 and MiR-135b-5p were associated with poor prognosis. On the other hand, high expressions of MiR-148a-3p, MiR-99a-5p, and let-7c-3p were found to be associated with better prognosis. Anal cancer is a rare disease with no public datasets on microRNAs available for confirmation of our findings. We therefore related our findings to published data from clinical- and preclinical studies from other cancer types as an indirect support.

In our series of anal cancer, no patient with low expression of MiR-1246 relapsed or died in contrast to the group where this miR was high. In SiHa squamous cell cervical carcinoma cultures, downregulation of MiR-1246 inhibited proliferation and tumour growth, increased apoptosis, blocked invasion in Matrigel, and caused cell cycle arrest in G1/S phase block at G1/S [20]. Higher tissue expression of MiR-1246 in cervical cancer, another HPV-induced squamous cell cancer, was similarly associated with lymph node metastases as we report for anal cancer [21]. Exosomal MiR-1246 induced cell motility and invasion also in an oral squamous cell carcinoma cell line [22]. In a lung cancer model, MiR-1246 target mRNA for GSK-3β and β-catenin, thus regulating the Wnt-pathway [23]. MiR-1246 has higher expression in malignant melanomas than normal tissues and is associated with invasion and metastasis [24]. In melanoma cells, MiR-1246 inhibited BAX but stimulated Bcl2, thus inhibiting apoptosis and hepatocyte nuclear factor 3-β/FOXA2 which is involved in embryonic development and activates liver genes. In colorectal cancer MiR-1246 promotes metastases via the MAPK pathway [25]. MiR-1246 is also a target for p53 [26]. For colon cells with gain of function mutations of p53 it is shown that the cells shed exosomes with MiR-1246 which stimulates macrophages to become type 2 macrophages stimulating the clinical growth of colon tumours [27]. Also, hypoxic glioma cells delivered exosomal MiR-1246 which induced M2 macrophages [28]. Exosomal MiR-1246 may be a smaller degradation product of a component of the spliceosome U2 (RNU2-1) in human cancer cells [29].

It is of special interest that exosomes containing MiR-1246 have been measured in serum and plasma from patients with cervical cancer [20], breast cancer [30, 31], hepatocellular cancer [32, 33], pancreatic cancer [34], gastric cancer [35], colorectal cancer [36], squamous cell esophageal carcinoma [37, 38], and prostate cancer [39]. Generally higher levels indicated poor prognosis.

MiR-135b-5p is upregulated in several gastrointestinal cancer types like gastric cancer [40, 41, 42], pancreatic cancer [43, 44], esophageal cancer [45], lymphomas [46], peripheral nerve sheath tumours [47], oral squamous cell cancers [48], and colon cancer [49, 50]. However, MiR 135b-5p is also reported to be lower in some tumours (i.e. pancreatic cancer) [51]. Its role may depend on the microenvironment, but most data support our finding of its role as an oncoMiR in anal cancer. Experimental data show further that miR-135b-5p targets the Wnt pathway through its inhibition of adenomatous polyposis coli (APC) [45, 46, 47, 49, 51] and Frizzled-1 [41], but also other targets like SFRP4 [42] and KRAS are reported [52].

In accordance with our findings in anal cancer patients, MiR-148a-3p has been reported as a tumour suppressor in several cancer types except osteogenic sarcoma and some gliomas [53]. This includes gastric cancer [54], oesophageal cancer [55, 56, 57], pancreatic cancer [58], non-small-cell lung cancer [59], laryngeal, and oral cancer [60, 61]. Low miR-148a-3p expression correlated with more aggressive features both *in vitro* and in hepatocellular patients where increased levels inhibited migration, invasion, and proliferation [62]. MiR-148a-3p targets known oncogenes and important signal pathways for tumour growth like Wnt and epithelial-mesenchymal transition (EMT) [58, 63], RAS-like protein1 (RALBP1) [60], DNMT1 [57, 61], c-Myc [63], SMAD2 [62], c-Met, snail, and other targets [64, 65]. MiR 148a-3p seems also to stimulate PD-L1 expression and low levels therefore contribute to environmental immunosuppression [66]. The presented data indicate a central role of miR-148a-3p in tumour growth.

Both the guide string miR-99a-5p and the complementary string miR-99a-3p were downregulated and associated with poor prognosis in head and neck squamous cell carcinomas [67, 68, 69], lung adenocarcinomas [70], breast cancer [71], and prostate cancer [72]. In poorly differentiated endometrial carcinoma tumours miR-99a-5p was downregulated and associated with reduced survival [73]. High serum levels of miR-99a-5p are presented as a possible positive biomarker in breast and gastric cancer [74, 75]. Several targets have been identified for this suppressor miR, that is, FAM64A, TIMP4, DNMT3B, and MCM4 [67, 70].

Let-7 is a family of MiRs discovered in 2001 which consists of 11 members [76, 77]. The let-7 family is downregulated in many cancer forms and reduced expression is associated with proliferation, invasion, and metastases and poor prognosis. The RNA-binding proteins LIN28A and LIN28B are direct targets of the let-7 family and are also inhibitors of let-7 biogenesis thus forming a double negative feedback loop [77]. A small molecule inhibitor of LIN28 increased let-7 and thereby reduced expression of PD-L1 and thus lowered immunosuppression in an experimental system [78]. Lin-7 also targets the high mobility group AT-Hook 2 (HMGA2), a transcriptional factor functioning as an oncogene [79, 80, 81], especially in less differentiated cancers [82]. Experimental studies further show that let-7 family members control cell cycle molecules and thereby proliferation [83]. In laryngeal cancer, let-7c-5p was downregulated in tumours and controlled the Pre-B-cell leukaemia homeobox transcription factor 3 (PBX3) [84]. In cervical cancer, the same miR was also identified as a tumour suppressing molecule controlling p16(INK4A) or CDKN2A, which are well-known factors in anal cancer [85]. In small cell cervical cancer let-7c is associated with more advanced tumour presentation and high expression is associated with very good prognosis in contrast to low expression [86]. Let-7c and MiR-99a cluster together at chromosome 21 of the human genome and their expressions have been shown to be similar in oral, esophageal, and bladder cancer [86, 87, 88, 89]. We also observed similar effects by these two microRNAs in our anal cancer patients. The proto-oncogene Myc which is an important regulator of many cellular processes, including proliferation, cell growth, metabolism, cell adhesion, motility, and angiogenesis, inhibits let-7c, which again inhibits Myc production in a feedback loop [90, 91, 92]. Let-7 stimulates degradation of PD-L1 and therefore suppresses immune suppression in head and neck squamous cell carcinomas and reduces survival [93].

Our study has several limitations like having a small number of tissue samples, and low number of recurrences and deaths due to anal cancer and lack of confirmation in independent analyses from other anal cancer cohorts and confirmation by laboratory tests. We cannot exclude that some of our identified microRNAs have a relation to the HPV status due to our limited sample size, especially since a relation to HPV is shown in the functional enrichment (Figure 3). However, the many reports of similar results in other cancer types support the findings of a prognostic clinical role in our study.

In conclusion, we have identified several oncoMiRs (miR-1246 and miR-135b-5p) and suppressor MiRs (miR-148a-3p, miR-99a-3p and Let-7c-3) as new potential prognostic factors in anal cancer patients. Hopefully, this report can stimulate more work that can confirm our first finding of MiRs as potential biomarkers in anal cancer.

## Ethics declaration

The study was conducted in accordance with the recommendations of The National Ethics Committee of Norway and Health Region West guidelines. All patients gave their written informed consent in accordance with the Declaration of Helsinki. The study was approved by the Regional Ethichs Committee IV (No 200/2000) and Regional Ethichs Committee Vest (Sak 5/6128).

## Data availability

The microRNA data supporting the findings of this study in anonymous form are available upon reasonable request. Genetic data, even in anonymous form, are restricted in Norway.
